# Virtual screening and evaluation of bioactive peptides from *Haliotis discus hannai* as potential HMGCR inhibitors for hyperlipidemia treatment

**DOI:** 10.3389/fnut.2024.1525768

**Published:** 2024-12-30

**Authors:** Kun Qiao, Lina Liu, Yihui Chen, Qiongmei Huang, Bei Chen, Jingna Wu, Wenmei Huang, Zhiyu Liu

**Affiliations:** ^1^Key Laboratory of Cultivation and High-Value Utilization of Marine Organisms in Fujian Province, Fisheries Research Institute of Fujian, Xiamen, China; ^2^College of Food Science, Fujian Agriculture and Forestry University, Fuzhou, China; ^3^Xiamen Medical College, Xiamen, Fujian, China; ^4^Xiamen Daozhiyuan Biological Technology Co., Ltd., Xiamen, China

**Keywords:** hyperlipidemia, 3-hydroxy-3-methylglutaryl-coenzyme a reductase, virtual screening, bioactive peptides, *Haliotis discus hannai*

## Abstract

**Introduction:**

Hyperlipidemia remains a major disease threatening global public health. The morbidity and mortality associated with cardiovascular diseases have been increasing. The inhibition of 3-Hydroxy-3-methylglutaryl-coenzyme A reductase (HMGCR), a key enzyme in the cholesterol synthesis pathway, can effectively reduce cholesterol levels.

**Methods and results:**

In this study, the most suitable protease for preparing HMGCR inhibitory peptides was screened using the evaluation indexes of peptide yield and HMGCR inhibition rate. Peptide sequences with molecular weights <1 kDa were identified, and peptide fragments were docked with HMGCR for virtual screening. The inhibitory effects of these peptides on HMGCR activity were evaluated *in vitro* using a high-fat Hep-G2 cell model. The screened peptides possessed significant HMGCR inhibitory activity and reduced cholesterol micelle solubility and total cholesterol and triglyceride levels in hyperlipidemic Hep-G2 cells.

**Conclusion:**

This study provides novel insights into developing natural drugs for hyperlipidemia; moreover, the results will facilitate the functional application of marine bioactive peptides.

## 1 Introduction

Hyperlipidemia, a major disease threatening global public health, is characterized by abnormal lipid metabolism, which results in significantly elevated cholesterol levels and other lipids in the blood (1). In recent years, the incidence of hyperlipidemia and the prevalence and mortality of associated cardiovascular diseases have been increasing, consequently attracting extensive research attention (2). In the pathological mechanism underlying hyperlipidemia, HMG-CoA reductase (HMGCR) is a key enzyme in the cholesterol synthesis pathway (3). Inhibiting HMGCR activity lowers cholesterol levels, helping to combat hyperlipidemia and its complications. The current clinical lipid-lowering drug atorvastatin is a competitive inhibitor of HMGCR. Although atorvastatin can effectively reduce lipid levels in patients with hyperlipidemia, certain side effects remain challenging (4). This necessitates novel HMGCR inhibitors, especially natural substances with higher safety (5).

In recent years, a growing body of research has indicated that natural active substances, such as proteins, peptides, and lipids, possess potential advantages in regulating blood lipids and may mitigate the risk of side effects for patients with hyperlipidemia (6, 7). These substances modulate lipid levels *in vivo* through multiple mechanisms, including the inhibition of cholesterol absorption, enhancement of cholesterol metabolism, and stimulation of lipoprotein lipase activity. Not only do these natural compounds effectively reduce total cholesterol (TC), low-density lipoprotein cholesterol (LDL-C), and triglycerides (TG), but they also elevate high-density lipoprotein cholesterol (HDL-C) levels, playing a constructive role in the prevention and treatment of cardiovascular diseases. For example, phytosterol ferulate significantly decrease cholesterol and lipid levels in the plasma and liver of mice fed a high-fat diet and promote the excretion of cholesterol in feces (8). With ongoing advancements in biotechnology and marine biology, marine bioactive peptides have drawn attention due to their distinct biological activities and potential applications. Collagen peptides derived from the skin of the *Sphyrna mokarran* downregulate the expression of FAS and HMGCR, while upregulating the hepatic expression of lecithin-cholesterol acyltransferase, thus alleviating cholesterol accumulation (9). Two pentapeptides, VIAPW and IRWWW, identified from the enzymatic hydrolysate of yellow croaker muscle, effectively reduce oleic acid-induced lipid accumulation in HepG2 cells and decrease intracellular levels of triglycerides and total cholesterol, exhibiting a significant dose–response effect. These peptides exert their hypolipidemic activity by downregulating the expression of lipid synthesis-related genes (SREBP-1c, SREBP-2, FAS, ACC, and HMGCR) and upregulating the expression of lipid oxidation-related genes (PPARα, ACOX-1, and CPT-1) (10). Marine bioactive peptides demonstrate substantial potential in modulating lipid metabolism and inhibiting HMGCR activity.

Despite the highly desirable advantages of marine bioactive peptides, their efficient preparation and purification face challenges. Conventional methods usually involve enzymatic digestion followed by separating the target peptides from the reaction mixture using suitable separation and purification techniques (e.g., gel column chromatography and electrophoresis) and validating the enzymatic products using analytical techniques (11). If further purification is required, chromatographic techniques [e.g., reverse-phase high-performance liquid chromatography (RP-HPLC) and ion-exchange chromatography] are usually employed, which are time-consuming and costly. Compared with the traditional experimental screening of a large number of compounds, virtual screening, as a computer-based technique, can rapidly screen compounds with potential biological activities by simulating the interactions between molecules and target proteins (12). Virtual screening efficiently predicts and evaluates the activity of various compounds and significantly reduces the time and cost of experimental screening. Thus, virtual screening has become an important tool in studying active marine peptides, improving research efficiency and reducing costs (13).

*Haliotis discus hannai* is the dominant species of abalone culture in China; its meat has high nutritional and medicinal value, making it a valuable seafood (14). Abalone, during processing, will produce a large amount of offal and other scraps; if not comprehensively utilized, it becomes a wasted resource (15). The visceral lipids, polysaccharides, and peptides of the wrinkled abalone have hypolipidemic effects, and antioxidant peptides and ACE inhibitory peptides originating from the skirt of the wrinkled abalone have been reported (16, 17). However, the development and utilization of visceral peptides from *Haliotis discus* have not been sufficiently explored. Therefore, this study aimed to identify the most suitable proteases for preparing HMGCR inhibitory peptides using peptide yield and HMGCR inhibition rate as evaluation indices and characterize the peptide sequences of peptide fractions with molecular weights <1 kDa. The inhibitory effect of these peptides on HMGCR activity was evaluated by virtual docking of these peptides with HMGCR, validation *in vitro*, and using a hyperlipidemic Hep-G2 cell model. The findings of this study will facilitate the development of natural drugs for hyperlipidemia and provide novel insights into the functional application of marine bioactive peptides.

## 2 Materials and methods

### 2.1 Chemicals and reagents

*Haliotis discus hannai* was purchased from Jinjiang Fuda Abalone Aquatic Products Co., Ltd. (Jinjiang, China). Pepsin and Papain were acquired from Nanning Pangbo Biological Engineering Co., Ltd. (Nanning, China). Acid Protease and Alkaline Protease were obtained from Beijing Solarbio Science & Technology Co., Ltd. (Beijing, China). Flavor Protease was supplied by Novozymes Biotechnology Co., Ltd. (Tianjin, China). Hep-G2 cells were obtained from the Kunming Cell Bank of the Kunming Institute of Zoology (Kunming, China). Fetal bovine serum was obtained from Cegrogen Biotech (Hessen, Germany). Penicillin/streptomycin and 0.25% trypsin solutions were obtained from ABK Biomedical (Halifax, Canada). Dulbecco’s Modified Eagle Medium (DMEM) and Dulbecco’s Phosphate-Buffered Saline (DPBS) were obtained from Gibco (Thermo Fisher Scientific, Waltham, MA, United States). Cell proliferation/cytotoxicity assay kits and 4% paraformaldehyde were obtained from Biosharp (Hefei, China). TC, TG, HDL-C and LDL-C assay kits were obtained from the Nanjing Jiancheng Bioengineering Institute (Nanjing, China). The abalone peptides were synthesized by GenScript (Nanjing, China).

### 2.2 Enzymatic digestion

Pepsin, acid protease, flavourzyme, papain, and alkaline protease were used to hydrolyze abalone viscera, respectively. The hydrolysis conditions are detailed in Supplementary Table 1. After hydrolysis, the samples were boiled for 15 min to deactivate the enzymes, cooled with running cold water, and then centrifuged at 4°C at 10,000 rpm for 30 min. The supernatant was aliquoted and stored at low temperature for future use.

After digestion, the enzyme was inactivated, and the enzyme digestion solution was obtained. The digest was centrifuged using a plate centrifuge and roughly filtered through a ceramic membrane. The filtrate obtained was ultrafiltered through a membrane of <1 kDa, and peptide fractions with a molecular weight of <1 kDa were collected and freeze-dried into powder.

### 2.3 Sequence identification

The polypeptide lyophilized powder was used for sequence identification, and a plurality of polypeptide sequences was obtained. The repeated sequences were deleted, and a plurality of non-repeated polypeptides was screened. Specifically, the lyophilized peptide powder was desalted using a C18 desalting column and analyzed by mass spectrometry (MS) using a mass spectrometer with a Nanojet ion source to obtain the peptide sequences. The entire system was a Q-Exactive Plus mass spectrometer (Thermo Fisher Scientific) in tandem with an EASY-nanoLC 1,200. The mass spectrometry conditions consist of a 0.1% formic acid in water for phase A, a 90% acetonitrile with 0.1% formic acid for phase B, operating in positive ion mode with electrospray ionization (ESI). A total of 1 μL of sample was uploaded (analytical column: Acclaim PepMap C18, 75 μm × 25 cm), and the samples were separated over 60 min with a controlled column flow rate of 300 nL/min, a column temperature of 40°C, and an electrospray voltage of 2 kV; the gradient was started from 2% of the B-phase, increased to 35% in a nonlinear gradient over 47 min, increased to 100% within 1 min, and maintained for 12 min. The mass spectrometer was operated in the data-dependent acquisition mode, automatically switching between MS and MS/MS acquisition. The MS parameters were as follows: MS: scanning range (m/z), 200–2,000; resolution, 70,000; AGC target, 3e6; maximal injection time, 50 ms. HCD-MS/MS: resolution, 17,500; AGC target, 1e5; maximum injection time, 45 ms; collision energy, 28; dynamic exclusion time, 30 s.

### 2.4 Determining the inhibitory effect of peptides on the micelle solubility of cholesterol

After sequence identification, the peptide sequences were obtained through solid-phase synthesis by GenScript (Nanjing, China). The inhibitory effect of the peptide on cholesterol micelle solubility was measured as previously described with some modifications (18). Micellar solutions (1 mL) contained 10 mM sodium taurocholate, 0.4 mM cholesterol, 1 mM oleic acid, 132 mM NaCl, and 15 mM sodium phosphate (pH 7.4). The peptide powder was dissolved in PBS to a final concentration of 10 mM and mixed with the cholesterol–micelle solution to prepare samples with final peptide concentrations of 50, 100, 200, and 400 μM. After mixing, the samples were ultrasonically emulsified at 37°C for 30 min and incubated on a shaker at 37°C for 24 h. The samples were centrifuged at 8,000 × *g* for 20 min, and a total cholesterol (T-CHO) assay kit (Jiancheng Bioengineering Institute, Nanjing, China) was used for measurement. The inhibitory effect of the peptide solution on cholesterol micelle solubility was calculated as follows:


(1) $$\begin{array}{l} \mathrm{Cholesterol micelle solubility inhibition rate}\;\mathrm{() =}\\ \;\frac{C_{0}-C_{1}}{C_{0}}\times 100 \end{array}$$

where C_0_ is the initial cholesterol concentration in the micelles, and C_1_ is the cholesterol concentration in the micelles after sample addition.

### 2.5 Determination of HMGCR activity

The HMG-CoA reductase inhibitory activities were determined using the HMG-CoA Reductase Assay Kit (Abcam, ab204701) as previously described (19). Enzyme activity was measured by monitoring the decrease in absorbance at 340 nm and 37°C, following the manufacturer’s instructions. The reaction mix consisted of a mixture of HMG-CoA:NADPH:HMG-CoA reductase buffer assay (12:4:174; 190 μL). In a 96-well flat-bottomed microtiter plate, 5 μL of HMG-CoA reductase, 2 μL of inhibitor, and 3 μL of buffer were mixed. The addition of 190 μL of reaction mix marked the initiation of the enzyme reaction. OD340 was measured for 10 min at 2-min intervals at 37°C.


$$\text{HMGCR inhibition rate (\%)}=\frac{\frac{-\Delta A\;340\;\mathrm{nm}}{\Delta T}\;\left(\mathrm{Enzyme}\right)-\frac{-\Delta A\;340\;\mathrm{nm}}{\Delta T}\;\left(\mathrm{Enzyme}+\mathrm{Inhubitor}\right)}{\frac{-\Delta A\;340\;\mathrm{nm}}{\Delta T}\;\left(\mathrm{Enzyme}\right)}\times 100.$$

### 2.6 Virtual screening

Protein HMGCR with the crystal structure 1HWK was used as the receptor, and the crystal structure of 3-hydroxy-3methyl-glutarate monoacyl-coenzyme A reductase (natural) was downloaded from the RCSB protein database (http://www.rcsb.org; PDB ID: 1HWK). The docking software Discovery Studio 2019 was used to hydrogenate and dehydrate the protein HMGCR molecules for energy minimization. The peptide molecules were mapped with Discovery Studio Client to draw small molecules as ligands. The active pockets of the HMGCR protein were identified and virtually screened under the conditions of Pocket Range 8 and Box Edge 4.

The results of virtual screening were ranked using the Grid Score, and based on the ranking and interactions, peptides with stronger binding interactions with the pockets (i.e., those with scores ranked close to the top) were selected for solid-phase synthesis, which was carried out at GenScript, to obtain the desired lipid-lowering peptides.

The ADMET prediction was conducted as per the following steps: (1) Create a 2D structure library of peptides in Discovery Studio 2019 software, Descriptors, and open the ADMET Descriptors dialog box. (2) Click the grid to the right of the Input Ligands, select pk_test: All in the dropdown list. (3) Choose to predict the properties for all small-molecule compounds, which can be checked to select the ADMET properties that need to be calculated; that is, select all ADMET properties.

### 2.7 Cell culture

Hep-G2 cells were cultured in DMEM supplemented with 10% fetal bovine serum and 1% penicillin/streptomycin. When the cells reached 80–90% confluency (log phase), they were enzymatically digested, gently mixed, centrifuged, and resuspended for passaging.

Log-phase cells were collected via centrifugation and diluted to 1 × 10^5^/mL. Then, 100 μL of the cell suspension was pipetted into a 96-well cell culture plate and cultured in a CO_2_ incubator for 14–16 h at 37°C until the cells reached 80–90% confluency. The cells were incubated with different peptide concentrations (50, 100, 200, and 400 μM) for 24 h, and the CCK8 assay kit was used to determine cell viability.

### 2.8 Determination of blood lipid levels

Cells grown to the log phase were digested, gently mixed using a pipette, and collected via centrifugation. The cells were then resuspended in a culture medium and homogenized using a pipette. A hemocytometer was used to count the cells diluted to 2 × 10^5^/mL using a complete medium. Then, 250 μL of the cell suspension was pipetted into a 48-well cell culture plate for incubation in a CO_2_ incubator at 37°C for 14–16 h. After reaching 80–90% confluency, cells were incubated with peptides (50, 100, 200, or 400 μM) and FFAs (10% oleic acid and palmitic acid) for 24 h. Control groups were performed without peptides or FFAs. The model groups were treated with FFAs only, without peptides. Next, the cells were washed with DPBS and lysed for 45 min on ice using 1.5% Triton-X100. TC, TG, HDL-C, and LDL-C levels were determined using Nanjing Jiancheng assay kits.

### 2.9 Molecular docking simulations

The X-ray crystal structure of 1HWK was obtained from the Protein Data Bank. The protonation state of the small molecule was set to pH = 7.4, and Open Babel (2.4.0) was used to generate the 3D structure of the compound. AutoDockTools (ADT3) was used to prepare and parameterize the receptor protein and ligands (20). After the docking grid box was generated using AutoGrid, molecular docking was performed using the AutoDock Vina software (version 1.2.0). The optimal protein–ligand conformation was used to analyze the interactions. Finally, PyMOL (2.5) was used to visualize the receptor–ligand interactions.

### 2.10 Molecular dynamics simulations

Molecular dynamics (MD) simulations were performed using the noncommercial version of Desmond/Maestro 2022.1 (21). The TIP3P water model was used to solvate the system, which was neutralized using a 0.15 M NaCl. After minimizing and relaxing the system, 100 ns MD simulations were performed in a 300-K, 1-bar isothermal, and isobaric ensemble. The MD trajectories were saved every 100 ps. The Simulation Interactions Diagram tool in Desmond was used for MD analyses.

### 2.11 Statistical analysis

The statistical analyses were conducted using SPSS 27.0 (SPSS, Chicago, IL, United States) software. When comparing the differences between the peptide treatment group and the control or model group, significance analysis was calculated by two-tailed independent sample t-tests. A difference of *p* < 0.05 was considered significant (*), a difference of *p* < 0.01 was consid-ered to be very significant (**). For comparing the inter-group differences among different peptide treatment groups, significance analysis was calculated by one-way ANOVA followed by a Tukey’s HSD *post-hoc* test and are indicated with different letters. Numbers (n) of samples or replicates are indicated in figure legends. For bar charts, all values are presented as mean ± s.d.

## 3 Results

### 3.1 Peptide yield and HMGCR inhibition rate of enzymatic hydrolysates

**Figure 1:**
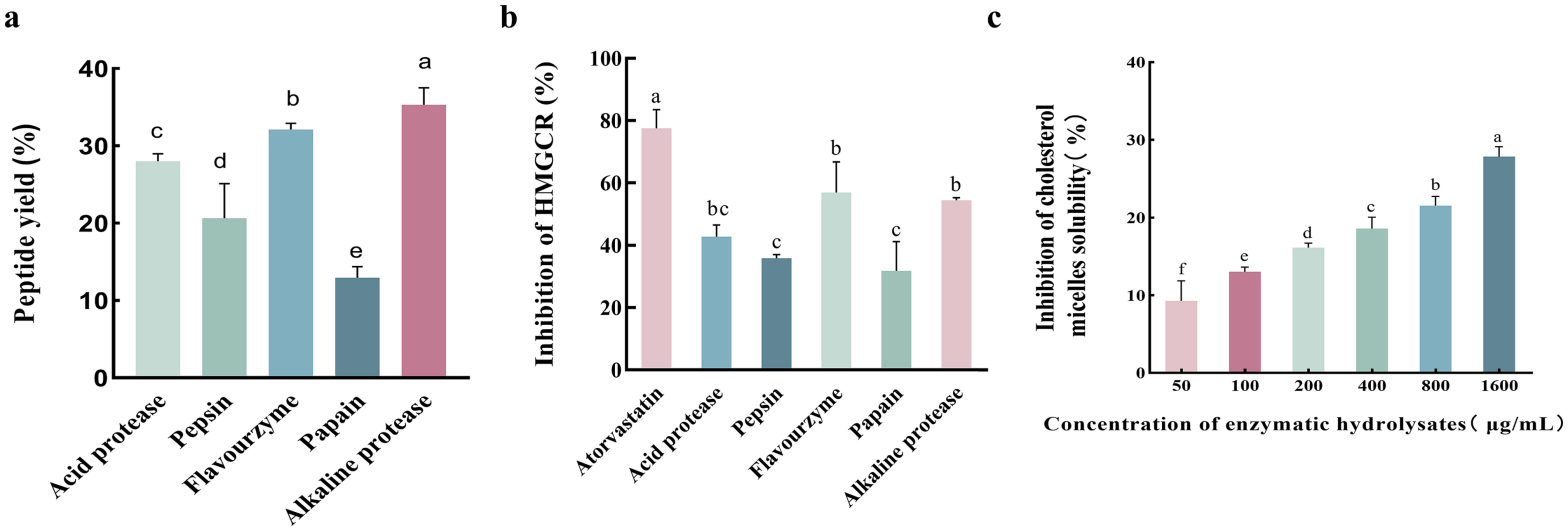
Comparative analysis of protease digestion products on peptide yield, HMGCR inhibition, and cholesterol micelle solubility inhibition. **(A)** Protein peptide yields of different protease digestion products. **(B)** HMGCR inhibition by different protease digestion products. **(C)** Inhibition of cholesterol micelle solubility of enzymatic hydrolysates *in vitro*. Lower case letters indicate significant differences between groups (*p* < 0.05). Error bars represent the SD. Mean values and SD are shown from three biological replicates.

Pepsin, acid protease, flavourzyme, papain, and alkaline protease were used to hydrolyze abalone viscera, respectively. The analysis of the peptide yield and HMGCR inhibition rate of the different protease digestion products in Figure 1 showed that the alkaline protease digestion product was superior to the other digestion products in terms of peptide yield and HMGCR inhibition rate. The peptide yield of the alkaline protease product reached 35.32% ± 1.57%, significantly different from that of other enzymatic products (*p* < 0.05, Figure 1A). Meanwhile, the HMGCR inhibition rate of the alkaline protease digestion product was second only to that of the positive control atorvastatin, with an inhibition rate of 54.45% ± 0.60% (Figure 1B). The *in vitro* cholesterol micelle solubility inhibition rates of the alkaline protease product are shown in Figure 1C. The micelle solubility inhibition rate increased significantly with increasing peptide concentration.

### 3.2 Virtual screening of small molecule active peptides targeting HMGR inhibition

**Figure 2:**
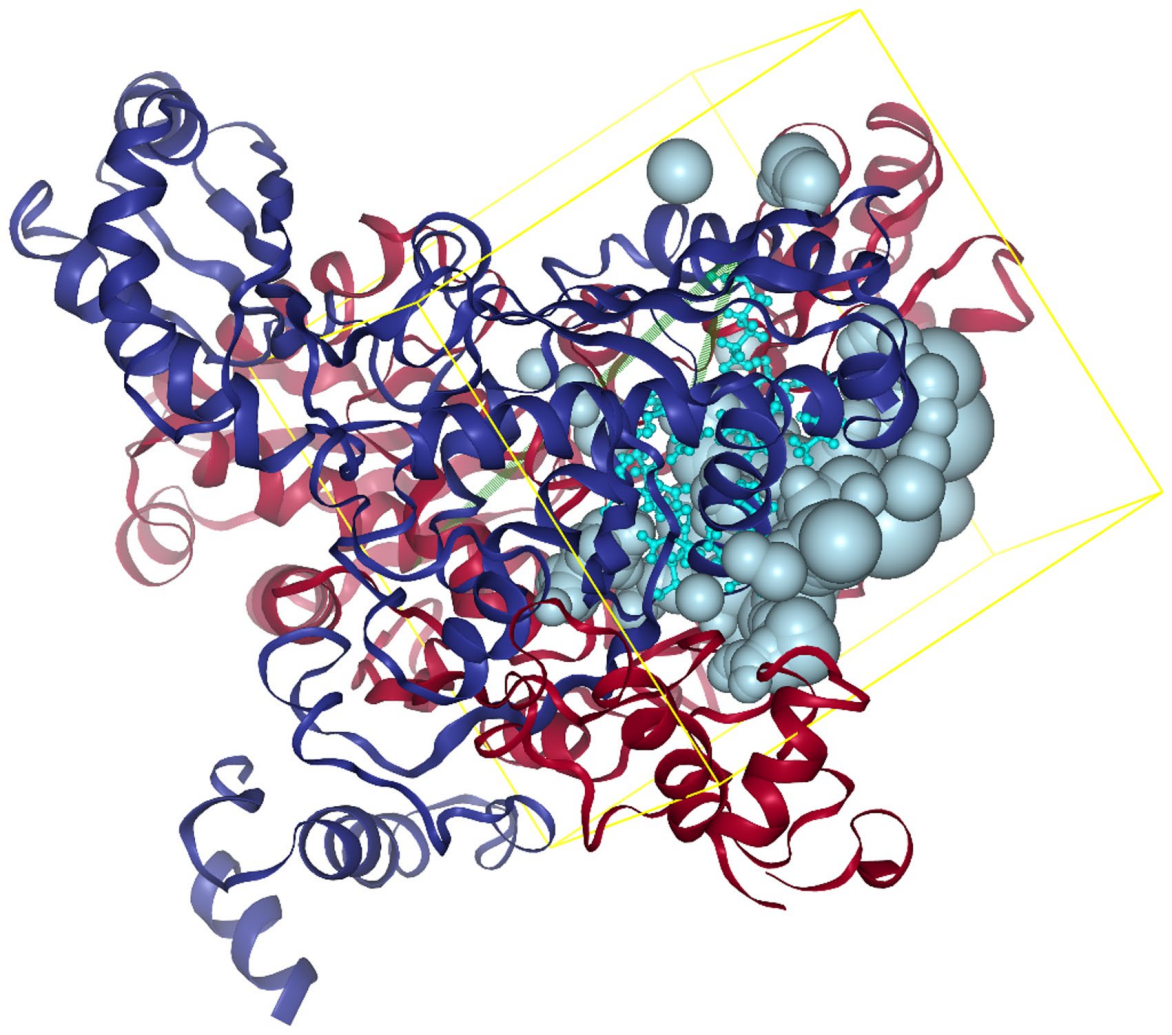
Schematic diagram of the HMGCR protein structure and box location. The yellow frame indicates the position of the HMGCR active site pocket, with the box center at (17.87, 3.41, 13.70) Å and the box dimensions at (40.71, 40.98, 38.70) Å.

SER463-GLY860; C chain: SER463-GLY860; C chain: LEU462-GLY860; D chain: SER463-GLY860. Referring to the method of Lin et al. (22), the C and D chains of 1HWK were retained, and the location of the active pocket of HMGCR was determined by defining amino acid residues in the following way: box center (17.87, 3.41, 13.70) Å; box size: (40.71, 40.98, 38.70) Å, as shown in Figure 2.

After identification and screening, 738 peptides were obtained with an amino acid number < 10, molecular weight < 1,000, and biological activity >0.75. The top 100 peptides were obtained according to Grid Scores and, after further virtual screening, ranked. The lower the Grid Score, the stronger the peptide binding to the receptor protein. The top 20 peptides with a number of hydrogen bonds of ≥2 and Grid-es of ≤ −2 kcal/mol were screened according to the Grid Score (Table 1). The ADMET prediction showed that none of the 20 peptides exhibited hepatotoxicity, Plasma Protein Binding (PPB) binding, oncogenicity, or CYP2D6 inhibition, indicating that they did not interfere with the normal *in vivo* action of the enzyme (Table 2). These peptides did not interfere with the normal activity of the enzyme (Table 2).

**Table 1:** Top 20 peptide sequences after virtual screening.

Number	Amino acid sequence	Mass	Number	Amino acid sequence	Mass
1	LMKFPMHPF	1147.46	11	KFPMHPF	903.10
2	WDWTKR	890.98	12	SDFSFK	729.77
3	IPGGIYPGR	929.07	13	SPADIGFH	842.89
4	PAYPMPWE	990.13	14	DAVMPNPF	890.02
5	APDMAFPR	904.05	15	GAPDMAFPR	961.10
6	QDAVMPNPF	1018.15	16	DYPRPW	833.10
7	WDWTRP	859.93	17	SYFFR	718.80
8	AYDPVFM	841.97	18	DFSFK	642.69
9	RYDPNPF	907.97	19	WFKTM	718.80
10	LPAYPMP	787.97	20	SPFFKTF	873.00

**Table 2:** ADMET prediction results for peptides.

	CYP2D6	Hepatotoxic	PPB	NTP
LMKFPMHPF	FALSE	FALSE	FALSE	Non-Carcinogen
WDWTKR	FALSE	FALSE	FALSE	Non-Carcinogen
IPGGIYPGR	FALSE	FALSE	FALSE	Non-Carcinogen
PAYPMPWE	FALSE	FALSE	FALSE	Non-Carcinogen
APDMAFPR	FALSE	FALSE	FALSE	Non-Carcinogen
QDAVMPNPF	FALSE	FALSE	FALSE	Non-Carcinogen
WDWTRP	FALSE	FALSE	FALSE	Non-Carcinogen
AYDPVFM	FALSE	FALSE	FALSE	Non-Carcinogen
RYDPNPF	FALSE	FALSE	FALSE	Non-Carcinogen
LPAYPMP	FALSE	FALSE	FALSE	Non-Carcinogen
KFPMHPF	FALSE	FALSE	FALSE	Non-Carcinogen
SDFSFK	FALSE	FALSE	FALSE	Non-Carcinogen
SPADIGFH	FALSE	FALSE	FALSE	Non-Carcinogen
DAVMPNPF	FALSE	FALSE	FALSE	Non-Carcinogen
GAPDMAFPR	FALSE	FALSE	FALSE	Non-Carcinogen
DYPRPW	FALSE	FALSE	FALSE	Non-Carcinogen
SYFFR	FALSE	FALSE	FALSE	Non-Carcinogen
DFSFK	FALSE	FALSE	FALSE	Non-Carcinogen
WFKTM	FALSE	FALSE	FALSE	Non-Carcinogen
SPFFKTF	FALSE	FALSE	FALSE	Non-Carcinogen

### 3.3 Effects of peptide fractions on TC content in high-fat Hep-G2 cells

**Figure 3:**
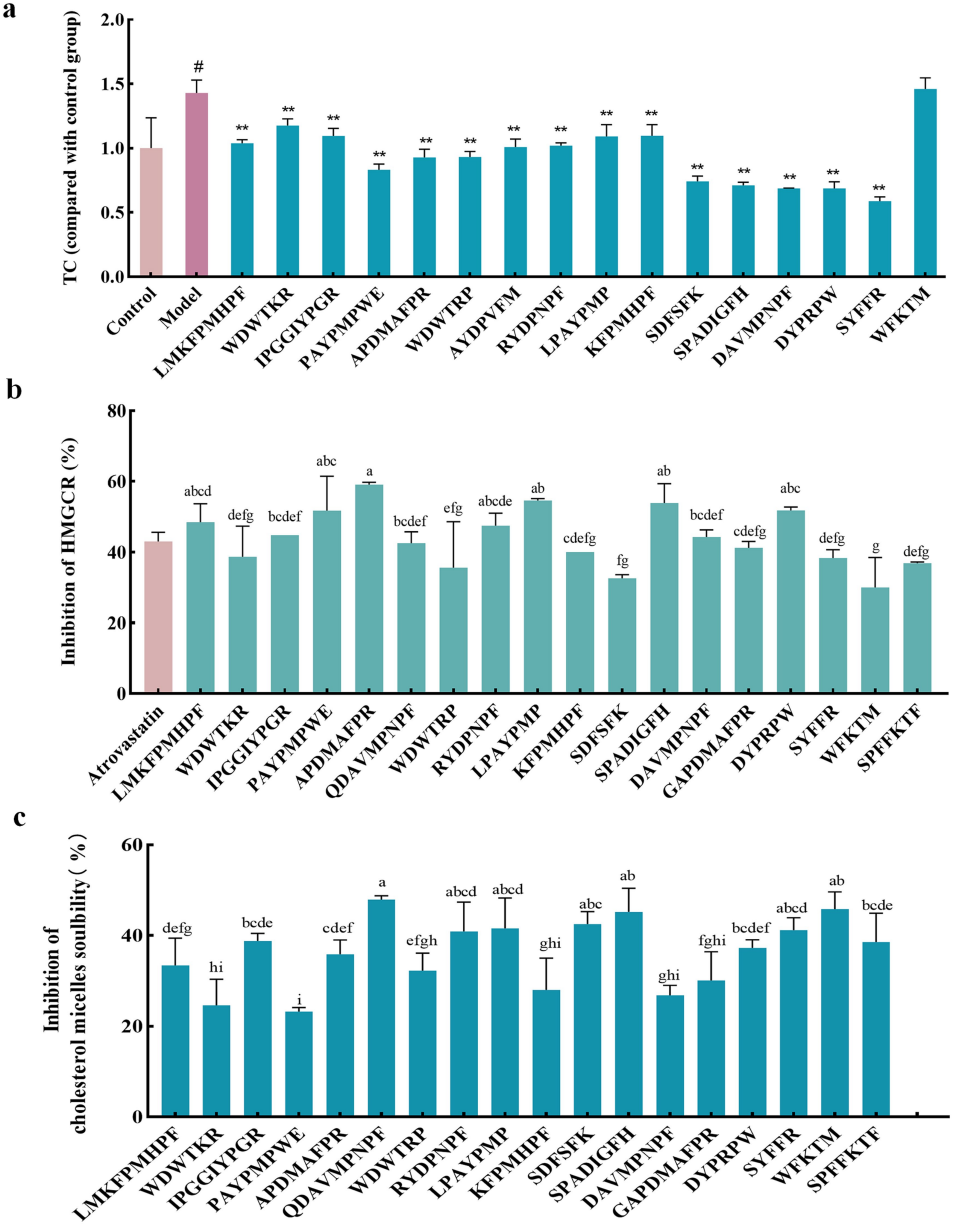
Evaluation of the hypolipidemic activity of different peptide fractions from Abalone. **(A)** Effect of peptide fractions on TC content in high-fat Hep-G2 cells. “*” indicates a significant difference between the treatment, model, and control groups (*p* < 0.05); “**” indicates a highly significant difference between the treatment, model, and control groups (*p* < 0.01); “#” indicates a significant difference between the treatment, model, and control groups (*p* < 0.05). **(B)** Inhibition rate of HMGCR by peptide fractions *in vitro*. Different letters indicate significant differences between groups (*p* < 0.05). **(C)** Inhibition of cholesterol micelle solubility of peptide fractions *in vitro.* Different letters indicate significant differences between groups (*p* < 0.05). Error bars represent the SD. Mean values and SD are shown from three biological replicates.

The liver is the primary site of endogenous cholesterol synthesis. To observe the effect of the peptide on cholesterol accumulation, FFAs-induced high-fat Hep-G2 cells were used as a test model, and the high-fat Hep-G2 cells were incubated with peptide fractions at 1 mM simultaneously. The accumulation of TC in the cells was measured 24 h later. The results showed that TC in high-fat Hep-G2 cells induced by FFAs in the model group significantly accumulated (*p* < 0.05) compared with that in the control group, and the TC content increased by 43.07% ± 0.17% (Figure 3A). In contrast, compared with the model group, incubation with all peptides, except for WFKTM, alleviated TC accumulation to a certain extent; however, the difference caused by the change in peptide concentration was not significant.

### 3.4 Inhibition rate of HMGCR by peptide fractions *in vitro*

HMGCR is a key rate-limiting enzyme in the cholesterol synthesis pathway and an important therapeutic target for lipid-lowering drugs. Inhibition of HMGCR inhibits cholesterol synthesis. The inhibition rate of HMGCR by 1 mM peptide was investigated using 0.5 mM Atorvastatin as a positive control. Figure 3B shows that these peptides had an inhibitory effect on HMGCR. Among them, the inhibitory effect of APDMAFPR, SPADIGFH, LPAYPMP, and DYPRPW was better than that of the positive control, with the inhibition rates of HMGCR reaching 59.55% ± 0.46, 53.89% ± 3.89, 54.65% ± 0.36, and 51.81% ± 0.70%, respectively.

### 3.5 Inhibition of cholesterol micelle solubility of peptide fractions *in vitro*

Cholesterol micelles simulated the cholesterol state of the small intestine; 1 mM of the peptide was added to the cholesterol micellar solution. If the peptide had an inhibitory effect on cholesterol micelle solubility, cholesterol was precipitated out of the micelle solution and centrifuged at the bottom of the tube. The inhibitory rate of the peptide’s cholesterol micelle solubility could be determined by detecting the amount of cholesterol in the supernatant. As shown in Figure 3C, the peptides inhibited the solubility of cholesterol micelles, among which the inhibition effects of QDAVMPNPF, SPADIGFH, APDMAFPR, LPAYPMP and DYPRPW were more obvious than those of other peptides, with inhibition rates of 47.94% ± 0.58, 45.23% ± 3.68, 35.87% ± 2.56, 41.55% ± 5.48, and 37.29% ± 1.45%, respectively.

By combining the effects of each peptide on TC accumulation in high-fat Hep-G2 cells and the inhibition of HMGCR and cholesterol micelle solubility *in vitro*, we found that APDMAFPR, QDAVMPNPF, LPAYPMP, and DYPRPW were outstanding in all three aspects.

### 3.6 Effect of abalone active peptides on TC and TG of Hep-G2 cells

**Figure 4:**
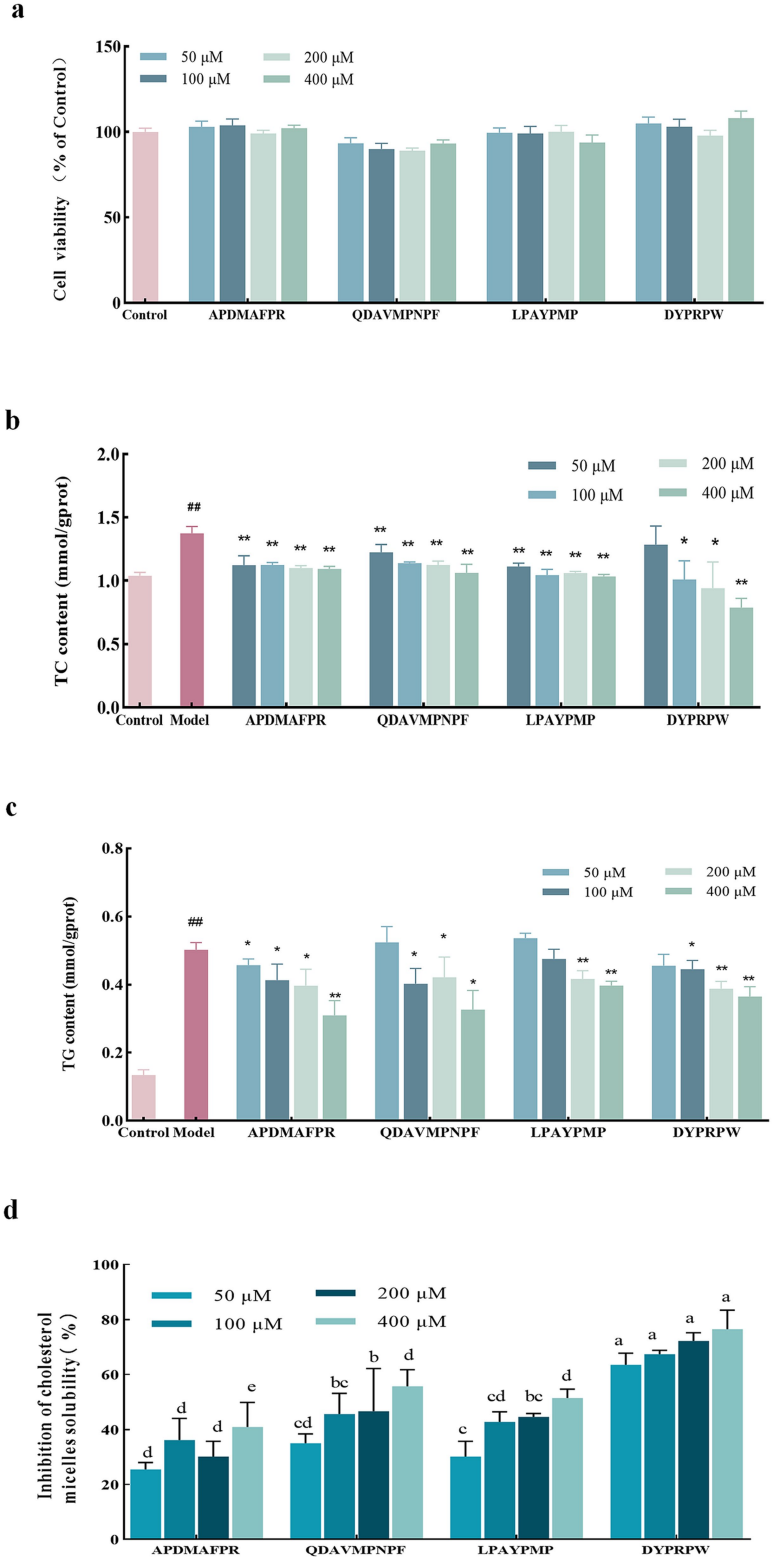
The impact of abalone active peptides at different concentrations on Hep-G2 cell lipids and cholesterol micelles. **(A)** The effect of varying concentrations of peptides on the cell viability of Hep-G2 cells. **(B)** The impact of varying concentrations of peptides on the TC content in high-fat Hep-G2 cells. **(C)** The influence of varying concentrations of peptides on the TG content in high-fat Hep-G2 cells. “*” indicates a significant difference between the treatment and model groups (*p* < 0.05); “**” indicates a highly significant difference between the treatment and model groups (*p* < 0.01); “#” indicates a significant difference (*p* < 0.05) between the control and model groups; “##” indicates a highly significant difference (*p* < 0.01) between the control and model groups. **(D)** Inhibition of cholesterol micellar solubility *in vitro* at different concentrations of abalone active peptides. Lower case letters indicate significant differences between groups (*p* < 0.05). Error bars represent the SD. Mean values and SD are shown from three biological replicates.

The results in Figure 4A show that none of the four peptides significantly affected Hep-G2 cell viability at the administered concentrations (*p* > 0.05), indicating that these four peptides were not cytotoxic at the selected concentrations and could be used in subsequent experiments. The FFA-induced Hep-G2 cell model was constructed. As shown in Figure 4B, compared with the model group, the four peptides at different concentrations alleviated the accumulation of TC in high-fat Hep-G2 cells, and the TC level in high-fat Hep-G2 cells showed a decreasing trend following DYPRPW treatment; the higher the concentration, the lower the TC level. The TC level in cells treated with 400 μM DYPRPW was significantly lower than that in the model group (*p* < 0.01) and close to that in the cells of the control group. The dose effects of APDMAFPR, QDAVMPNPF, and LPAYPMP peptide treatments were not significant.

High-fat Hep-G2 cells were used as a model, and the TG content was determined. As shown in Figure 4C, TG accumulation induced by FFAs was significant. The TG content in the cells of the model group reached 3.75 ± 0.45 times that in the cells of the control group. Meanwhile, the four peptide treatments at different concentrations reduced the TG levels in high-fat Hep-G2 cells; however, except for the APDMAFPR treatment, none of the other three peptide treatments at a concentration of 50 μM induced a reduction in TG levels. The large accumulation of TG induced by modeling possibly resulted in minimal changes from peptide incubation at low concentrations. In contrast, the TG content of APDMAFPR and QDAVMPNPF treated at 400 μM was significantly lower than that of the model group, reaching 0.31 ± 0.04 and 0.33 ± 0.05 mmol/gprot, respectively, with a decrease of 34.86% ± 0.08 and 31.31% ± 0.10%, compared with that in the model group, respectively.

### 3.7 Effects of abalone active peptides on *in vitro* cholesterol micellar solubility inhibition rate

The *in vitro* inhibition rates of cholesterol micellar solubility at (Equation 1) different concentrations of the four peptides are shown in Figure 4D. DYPRPW showed a superior inhibition rate of *in vitro* cholesterol micellar solubility to the other peptides, and the inhibition rates of the two peptides were the highest at a treatment concentration of 400 μM, reaching 76.50% ± 5.62%. QDAVMPNPF and LPAYPMP also showed inhibition of cholesterol micellar solubility. The higher the treatment concentration, the more pronounced the inhibitory effect. APDMAFPR showed the weakest inhibitory effect compared with the other three peptides.

### 3.8 HMGCR enzyme inhibition rate of abalone active peptides

**Figure 5:**
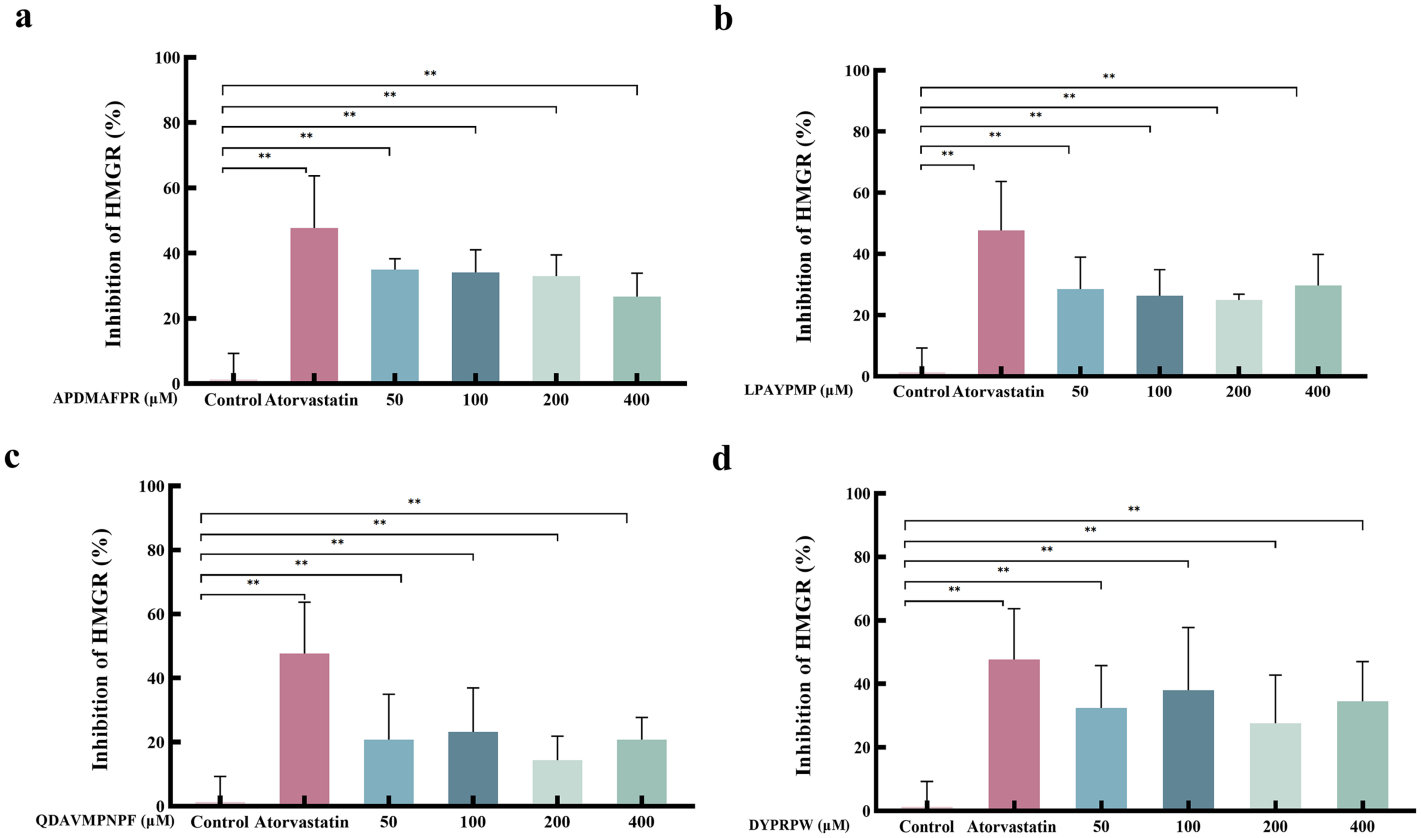
Effect of different concentrations of abalone active peptides on HMGCR enzyme activity *in vitro.* **(A)** HMGCR inhibitory activity of APDMAFPR. **(B)** HMGCR inhibitory activity of LPAYPMP. **(C)** HMGCR inhibitory activity of QDAVMPNPF. **(D)** HMGCR inhibitory activity of DYPRPW. Error bars represent the SD. Mean values and SD are shown from three biological replicates. “*” indicates a significant difference between the treatment and control groups (*p* < 0.05); “**” indicates a highly significant difference between the treatment and control groups (*p* < 0.01).

Combining the results of the above peptide fractions, four abalone-active peptides (APDMAFPR, LPAYPMP, QDAVMPNPF, and DYPRPW) were selected for further follow-up experimental studies. Figure 5 shows the effect of different concentrations of abalone active peptides on HMGCR enzyme activity *in vitro*. HMGCR inhibitory activity is shown for all four peptides at concentrations of 50–400 μM.

### 3.9 Molecular docking force between abalone active peptides and HMGCR

**Figure 6:**
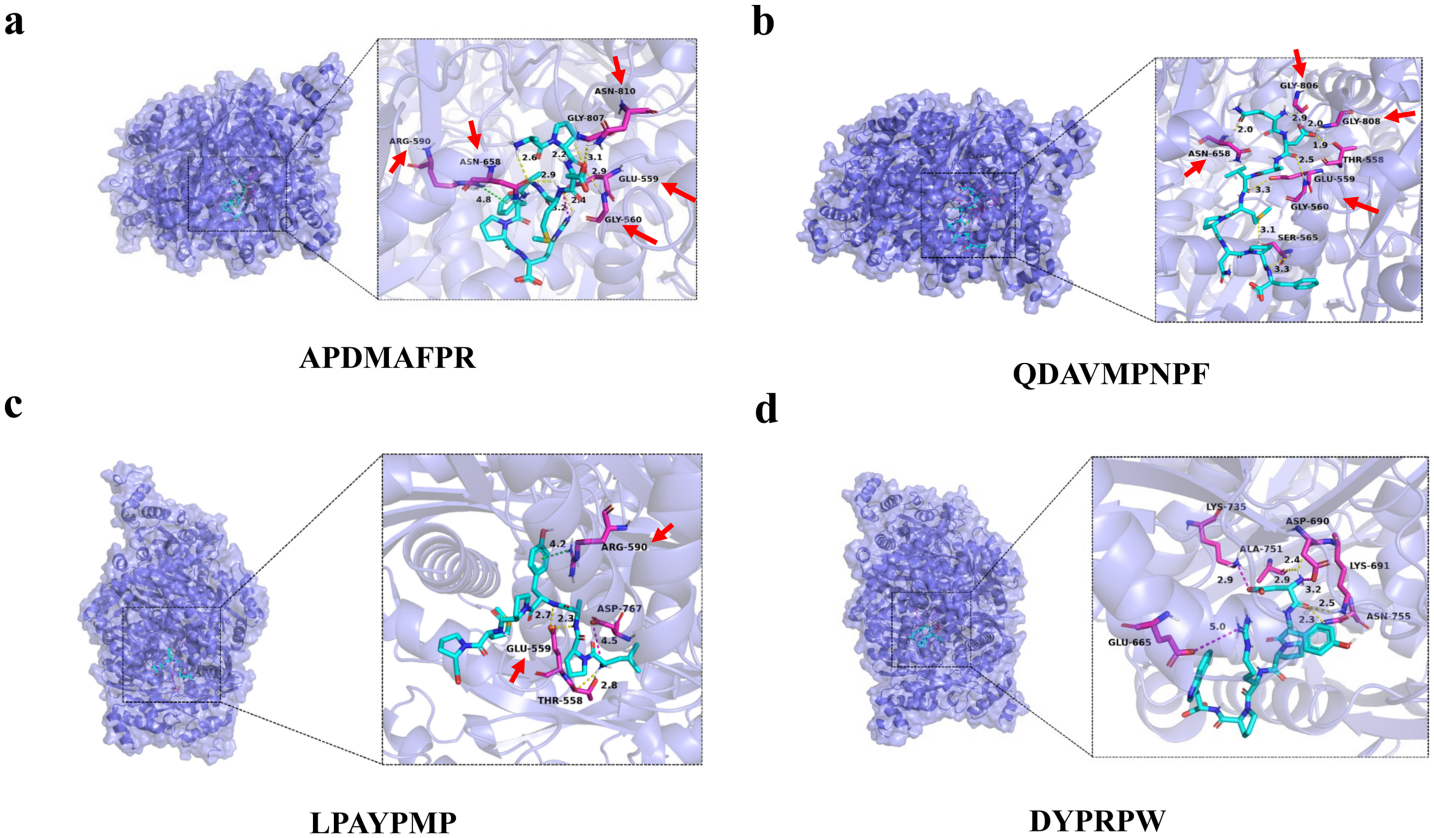
Molecular docking diagram of abalone active peptides with 1HWK. **(A)** Force diagram of APDMAFPR vs. HMGCR; **(B)** force diagram of QDAVMPNPF vs. HMGCR; **(C)** force diagram of LPAYPMP vs. HMGCR; and **(D)** force diagram of DYPRPW vs. HMGCR.

To verify the binding ability of the lipid-lowering peptide and HMGCR, molecular docking was used to simulate and analyze the force between the two peptides, using HMGCR as the receptor protein and the four peptides as ligands. The results are presented in Figure 6 and Table 3.

**Table 3:** Abalone active peptides and 1HWK interaction force analysis.

Receptor	Ligand	Score	RMSD (Å)
1HWK	APDMAFPR	−8.1 kcal/mol	Glu 559(2.4 Å); Gly 560(2.9 Å); Asn 658(2.6 Å, 2.9 Å); Asn 810(3.1 Å); Glu 559(3.2 Å); Arg 590(4.8 Å)
	QDAVMPNPF	−8.1 kcal/mol	Gly 806(2.9 Å); Asn 658(2.0 Å); Ser 565(3.1 Å, 3.3 Å); Gly560(2.5 Å); Glu 559(3.3 Å); Thr 558(1.9 Å); Gly 808(2.0 Å);
	LPAYPMP	−8.2 kcal/mol	Ser 565(2.1 Å); Gly 560(2.2 Å); Gly 860(2.2 Å, 2.4 Å); Glu 665(4.7 Å)
	DYPRPW	−8.2 kcal/mol	Thr 558(2.8 Å); Glu 559(2.3 Å, 2.7 Å); Asp 767(4.5 Å); Arg 590(4.2 Å)

Å is a unit that indicates the size of the area of the protein interaction surface. Yellow indicates that the mode of force is hydrogen bonding, green indicates hydrophobic interaction, and fuchsia indicates electrostatic interaction.

The amino acid residues GLU559, ASN658, GLY807, and GLY560 of 1HWK formed hydrogen bonds with the ligand APDMAFPR; GLU559 formed electrostatic interactions with the ligand, while ARG590 formed hydrophobic interactions. The binding energy of the protein-small molecule complex was −8.1 kcal/mol. The amino acid residues GLY806, GLY560, GLY808, and GLY560 of 1HWK formed hydrogen bonds with the ligand QDAVMPNPF. The binding energy of the protein-small molecule complex was −8.1 kcal/mol. The amino acid residues THR558 and GLU559 of 1HWK formed hydrogen bonds with the LPAYPMP ligand, and ARG590 formed hydrophobic interactions with the ligand; the binding energy of the complex was −8.2 kcal/mol. The amino acid residues LYS691 and ASP690 of 1HWK and ALA751 formed hydrogen bonds with the ligand DYPRPW, while GLU665 and ASP690 formed electrostatic interactions; the binding energy of the complex was −8.2 kcal/mol.

Taken together, the binding energies of the complexes formed by 1HWK and the four ligands were good, and the amino acid residues near the active pocket of the receptor proteins, such as GLU559, GLY560, ARG590, and ASP690, played a major role. Hydrogen bonding plays a major role in forming the binding forces, followed by electrostatic and hydrophobic interactions. Hydrogen bonds are more stable, and the higher the number of hydrogen bonds, the more stable the complex structure.

### 3.10 Stability analysis of abalone active peptide pair binding to HMGCR

In MD simulations, RMSD is a commonly used metric for assessing simulation results and comparing the degree of difference between the atomic or molecular structures of a simulated system and reference structure. RMSD provides information on the overall stability and degree of variation of the simulated system structure with respect to the reference structure. A low RMSD value indicates a small structural difference between the simulated system and reference structure; hence, the simulation results maintain the stability of the reference structure well (23). In contrast, a higher RMSD value indicates a larger structural difference between the simulated and reference structures.

**Figure 7:**
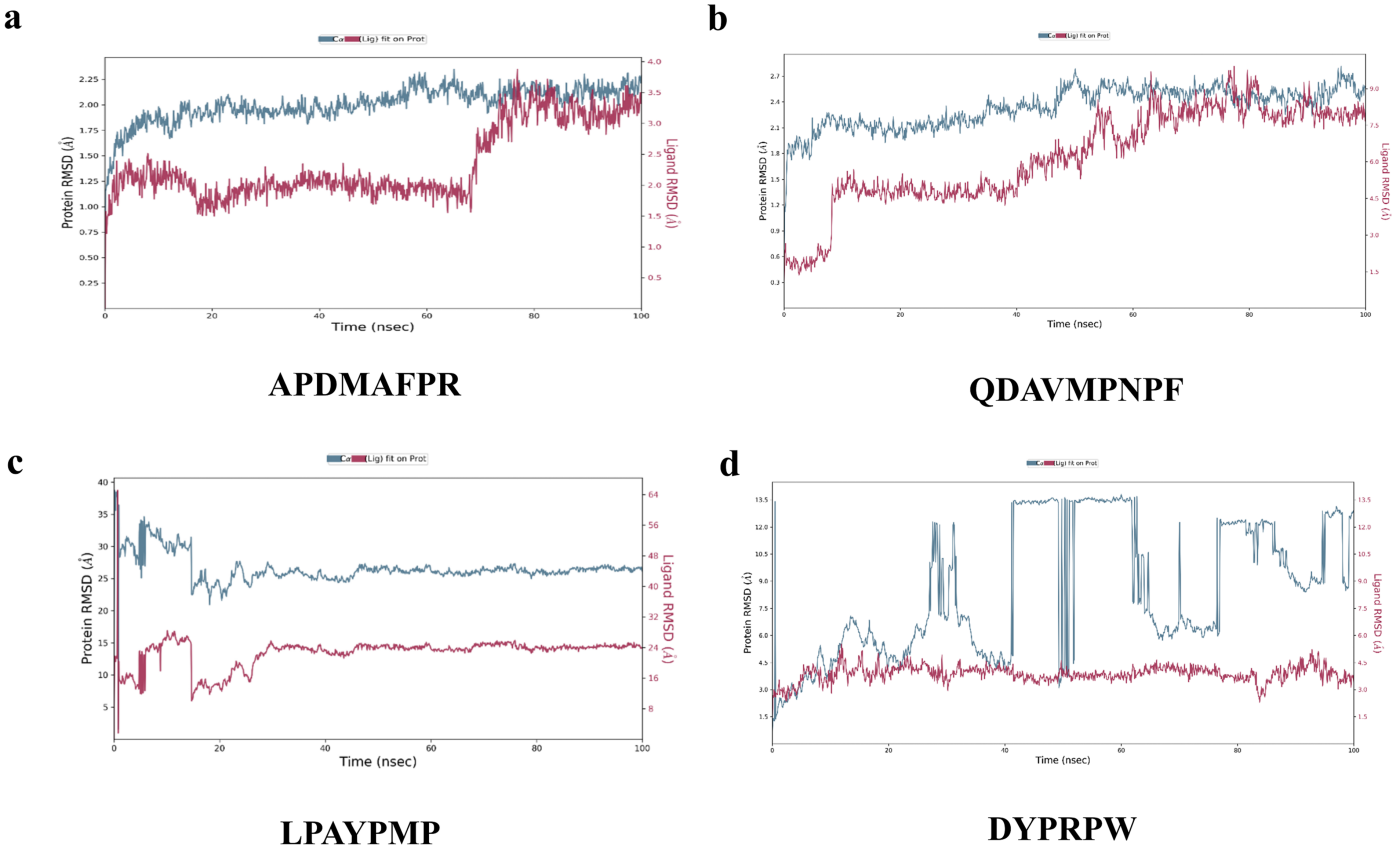
Changes in RMSD values in molecular dynamics simulations of peptides with 1HWK. **(A)** APDMAFPR vs. HMGCR; **(B)** QDAVMPNPF vs. HMGC; **(C)** LPAYPMP vs. HMGCR; **(D)** DYPRPW vs. HMGCR. The left y-axis denotes the evolution of the RMSD of the protein, while the right y-axis denotes the stability of the ligand relative to the protein and its binding pocket.

The results of the MD simulations of APDMAFPR and HMGCR are shown in Figure 7A. During the simulation process, the RMSD of the protein was stabilized in the range of 1.75–2.25 Å, while the final RMSD of the ligand APDMAFPR was stabilized in the range of 3.0–3.5 Å following fluctuation. This indicates that the initial conformations of the two ligands have only small fluctuations relative to the initial conformations, suggesting that their initial conformations have a certain degree of stability and that a more stable binding conformation is formed based on the original conformation.

The results of the MD simulations of QDAVMPNPF and HMGCR are shown in Figure 7B. During the simulation process, the RMSD of the protein was stabilized in the range of 2.1–2.7 Å, while the final RMSD of the ligand was stabilized between 7.5 and 9.0 Å following fluctuation. This indicates that the initial conformations of the two molecules changed considerably, although their final binding conformations were relatively stable. The results indicate that the initial conformation of the small molecule and protein binding are not stable; however, after kinetic simulation, the two form a new, more stable conformation and have a strong binding force in this conformation.

Figure 7C shows the results of the MD simulations of LPAYPMP and HMGCR. During the simulation, the protein’s RMSD fluctuated and stabilized at ~45 Å, while the ligand’s RMSD fluctuated and stabilized at ~24 Å. These results indicate that the binding of small molecules and proteins was not stable in the initial conformation. Moreover, the initial conformation of the small molecule–protein binding was unstable, and the small molecule moved away from the binding pocket during simulation.

The results of the MD simulations of DYPRPW and HMGCR are shown in Figure 7C. The protein significantly fluctuated during the simulation, and its RMSD was not stabilized. In contrast, the ligand fluctuated and stabilized its RMSD at ~3.5 Å. This suggests that the initial conformations of the two molecules were not stable, indicating that the initial conformations of the two proteins were stable. The larger protein fluctuation may be because the tetrameric protein is larger and other protein parts are less stable.

In summary, APDMAFPR, QDAVMPNPF, and HMGCR were more stable, and the ligand conformations did not significantly change during the kinetic simulation, whereas LPAYPMP, DYPRPW, and HMGCR either had poorer stability or the ligand conformations significantly changed during simulation, which deviated from the pocket position.

## 4 Discussion

The association between hyperlipidemia and cardiovascular diseases has been well recognized, and HMGCR, a key enzyme in the cholesterol synthesis pathway, remains a major target for treating hyperlipidemia. The activities of natural compoundsvities have potential advantages in regulating blood lipids and may reduce the risk of side effects in patients with hyperlipidemia. These naturally active substances include polyphenols, flavonoids, phytosterols, dietary fibers, and unsaturated fatty acids (24–27), which regulate lipid levels *in vivo* through various mechanisms, such as the inhibition of cholesterol absorption, promotion of cholesterol metabolism, and enhancement of lipoprotein esterase activity. These natural substances not only effectively reduce TC, LDL-C, and TG but also increase the level of HDL-C, playing a positive role in the prevention and treatment of cardiovascular diseases. In this study, wrinkled disk abalone was used as a raw material to screen for peptides with significant HMGCR inhibitory activity by targeting HMGCR; their conformational relationships were investigated, revealing their potential to inhibit HMGCR activity. This provides a new strategy for the development of natural drugs for the treatment of hyperlipidemia.

Protease digestion is a common means of preparing bioactive peptides. Whereas protease enzymatic sites are specific, previous studies found that the activity is linked to the composition of amino acids and the structure of peptides. Therefore, the selection of appropriate proteases is vital for the preparation of active peptides (28). Alkaline protease has strong alkali, heat, and hydrolysis resistance; this experiment showed that alkaline protease enzymatic hydrolysis products have higher peptide yield than that of the enzymatic hydrolysis products of other proteases. The enzymatic hydrolysate of Zosterissessor ophiocephalus, under the action of alkaline protease, is rich in glycine (GLY). This substance has been found to reduce the enzymatic activity of HMGCR in the serum of high-fat diet (HFD) rats and downregulate the expression of LDLR, thereby hindering the synthesis of cholesterol within the rats’ bodies (29). Hypolipidemic active peptides were isolated, purified, and identified in all the enzymatic products of chickpea flour digested by alkaline protease (30). Similarly, the results of the present experiment showed that alkaline protease enzymatic products showed higher HMGCR inhibition as compared with the enzymatic products of other proteases. Proteins with molecular weight < 1 kDa are usually more readily absorbed. This is because, in the gut, smaller proteins can be absorbed more readily through cell gaps or cell membranes, whereas large ones may be restricted (31). The strong bioactive function of peptides with smaller molecular weights may be related to their easy access to target organs to participate in the regulation of physiological metabolism (32).

Gomes et al. (33) simulated the gastrointestinal digestion of proteins from cauliflower beans (*Phaseolus vulgaris L*) with pepsin and trypsin to obtain peptides with molecular weights <1 kDa (LVTTTVDL, QTSTPLFS, VELVGPK, and TRGVLV) and found that the peptides, in addition to their cholesterolemia-lowering effect, also prevented inflammation and dysfunction of the vascular endothelium in addition to reducing oxidative stress. Therefore, in the present study, peptide fractions with molecular weight < 1 kDa were obtained by ultrafiltration membrane for sequence identification. Prados et al. (34) isolated a hypolipidemic peptide from the product of alkaline protease digestion of olive seeds. The results showed that acidic amino acids such as GLU, GLN, ASP, and ASN play a role in hypolipidemia; therefore, acidic amino acids are a common feature of hypolipidemic peptides. In this study, the hypolipidemic peptides of abalone, such as QDAVMPNPF, were virtually screened out, consistent with our previous findings.

The protein structure and catalytic domains of HMGCR are well-defined, and peptides with strong binding to HMGCR were obtained after virtual screening and scoring using HMGCR as the receptor protein, which can predict peptide properties, including absorption, distribution, metabolism, water solubility, and toxicity. CYP2D6, also known as cytochrome P4502D6, is primarily involved in drug metabolism. The inhibition of CYP2D6 interferes with or reduces the metabolism of drugs by this enzyme. Compounds can affect their clearance rate and plasma concentration by inhibiting CYP2D6, which may cause drug–drug interactions, increase potential drug toxicity, or alter drug efficacy (35). Therefore, understanding and evaluating drugs’ inhibitory properties against CYP2D6 during drug therapy is crucial to avoid adverse drug reactions and enhance drug efficacy. The liver has important detoxification, metabolism, and storage functions. Hepatotoxicity can cause liver damage or injury, affecting the body’s metabolism and detoxification capacity. PPB refers to the degree of drug binding to proteins in the plasma. The plasma protein binding rate is important for determining drug dosage, interactions, and safety. It has been found that proteins in the plasma can form non-covalent bonds with drugs, affecting drug distribution and metabolism, and that drugs bound to proteins are not easily metabolized by the liver, slowing drug elimination (36). In the present study, the top 20 peptides in the virtual screening binding score had good transport properties and were non-carcinogenic. Iqbal et al. (37) isolated and purified bioactive compounds derived from *Ficus virens Ait* extracts, performed *in vitro* HMGCR inhibition and enzyme kinetic assays using HMG-CoA as a substrate, and rapidly screened 1 of 20 new compounds for HMGCR antioxidant activity. HMGCR inhibitors with antioxidant activity were screened from the 20 new compounds, and HMGCR enzyme-type inhibition and pharmacokinetic data of the new compounds were evaluated via molecular docking. Lin et al. (22) used the 3D structure of 1HWK in the PDB database of HMGCR as a target and screened approximately 30,000 pure compounds from a database of traditional Chinese medicines (TCM), including polyphenolic compounds, poly substituted heterocyclic compounds, and linear lipophilic alcohols. A total of 30,000 pure compounds from the TCM database (including polysubstituted heterocyclic compounds and linear lipophilic alcohols) were used to screen 561 compounds with scores comparable with those of the positive control.

By analyzing the binding force of the four abalone active peptides with 1HWK, we illustrated the role of hydrogen bonding in protein–ligand interactions. Amino acid residues around the active pocket of the receptor protein, such as GLU559, GLY560, ARG590, and ASP690, are more likely to bind to lipid-lowering peptides. Jamuna et al. (38) found that the amino acid residues interacting with procyanidins were CYS561, ARG568, HIS861, HIS722, ASN567, HIS568, HIS568, HIS861, and HIS861, using HMGCR (PDB:1DQ9) as the receptor protein. LYS722, ASN567, SER852, ALA564, LYS864, SER565, and atorvastatin-interacting amino acid residues included CYS561, ALA564, ARG568, ASN567, LYS722, HIS861, and LYS864. Aqeel et al. (39) used 1HWK as a receptor and, after screening eight compounds, revealed that one compound showed better binding affinity (−8.3 and − 7.9 kcal/mol), indicating that the compound interacted with VAL80, MET656, THR558, and GLU559 through hydrogen bonding at the HMGCR active binding site. This suggests that the binding site and force of the peptide on the target protein HMGCR in this study were similar to those of atorvastatin and other lipid-lowering compounds and that enzyme inhibitory activity was achieved by occupying the binding site of the substrate. The results of the molecular binding force analysis suggest that the abalone active peptide can potentially be an HMGCR.

The *in vitro* results and the hyperlipidemic Hep-G2 cell model in this study showed that the screened peptides possessed significant HMGCR inhibitory activity, which could reduce cholesterol micellar solubility and decrease TC and TG content in cells. Moreover, the cytotoxicity results suggest a lower risk of side effects of the peptides. This provides a new natural drug option for treating hyperlipidemia and suggests that these peptides have potential applications for lowering lipid levels. Furthermore, *in vivo* experiments and clinical trials remain warranted to validate the safety and efficacy of these peptides. Future studies should focus on optimizing the structure of peptides, improving their stability and bioavailability, and exploring their pharmacokinetic properties *in vivo* to facilitate their translation into clinical applications.

## 5 Conclusion

In this study, we successfully screened peptides that effectively inhibited the activity of HMGCR, providing novel natural drug candidates for treating hyperlipidemia. Using a combination of protease screening, peptide sequence identification, virtual screening, *in vitro* experiments, and cell model evaluations, we confirmed the inhibitory effects of these peptides on HMGCR at the molecular and cellular levels and their potential to lower cholesterol levels. These findings indicate that marine bioactive peptides can potentially act as HMGCR inhibitors and provide a new strategy for developing novel cholesterol-lowering drugs.

## Data Availability

The original contributions presented in the study are included in the article/Supplementary material, further inquiries can be directed to the corresponding author.
