# Supplementary material for: Virtual screening and evaluation of bioactive peptides from Haliotis discus hannai as potential HMGCR inhibitors for hyperlipidemia treatment
Source: Front Nutr. 2024 Dec 30;11:1525768. doi: 10.3389/fnut.2024.1525768 (PMC11730078; doi:10.3389/fnut.2024.1525768)
Supplement: Supplementary file 1 [file Table_1.DOCX]

Supplementary Materials

Table 1. Enzymatic conditions for five proteases

|  | Enzyme (U/g) | Hydrolysis Temperature (℃) | Hydrolysis Time (h) | Hydrolysis  pH |
| --- | --- | --- | --- | --- |
| Acid protease | 10000 | 35 | 4 | 3.0 |
| Pepsin | 10000 | 35 | 4 | 3.0 |
| Flavourzyme | 10000 | 50 | 4 | 7.5 |
| Papain | 10000 | 50 | 4 | 7.5 |
| Alkaline protease | 10000 | 55 | 4 | 10.0 |
